# 

**Published:** 1891-03

**Authors:** 


VOL. XII. MARCH, 1891. No. 3.

LIBRARY
Indexed
S. G. O.

### THE
### International Dental Journal

A MONTHLY PERIODICAL

DEVOTED TO

### DENTAL AND ORAL SCIENCE.

**JAMES TRUMAN, D.D.S., Editor.**

Publication Office,
716 FILBERT STREET, PHILADELPHIA.

PUBLISHED BY THE
INTERNATIONAL DENTAL PUBLICATION COMPANY,
51 WEST THIRTY-SEVENTH STREET, NEW YORK CITY,
—AND—
716 FILBERT STREET, PHILADELPHIA.

ENTERED AT PHILADELPHIA POST-OFFICE AS SECOND-CLASS READING MATTER.

$2.50 per Annum, in Advance. Single Copies, 25 Cents.

PRINTED BY J. B. LIPPINCOTT COMPANY, PHILADELPHIA.

